# Comparison of Pre-Endoscopic C-WATCH Score with Established Risk Assessment Tools in Patients with Upper Gastrointestinal Bleeding

**DOI:** 10.1159/000522121

**Published:** 2022-01-24

**Authors:** Gabriel Allo, Martin Bürger, Johannes Gillessen, Philipp Kasper, Jeremy Franklin, Vera Mück, Dirk Nierhoff, Hans-Michael Steffen, Tobias Goeser, Christoph Schramm

**Affiliations:** ^a^Department of Gastroenterology and Hepatology, Faculty of Medicine and University Hospital Cologne, University of Cologne, Cologne, Germany; ^b^Institute of Medical Statistics and Computational Biology, University of Cologne, Cologne, Germany; ^c^Clinic for Gastroenterology, Hepatology and Transplant Medicine, Essen University Hospital, Essen, Germany

**Keywords:** Gastrointestinal bleeding, Mortality, C-WATCH score, Rockall score, Glasgow-Blatchford score

## Abstract

**Introduction:**

Use of risk scores for early assessment of patients with upper gastrointestinal bleeding (UGIB) is recommended by various guidelines. We compared Cologne-WATCH (C-WATCH) score with Glasgow-Blatchford score (GBS), Rockall score (RS), and pre-endoscopic RS (p-RS).

**Methods:**

Patients with UGIB between January and December 2017 were retrospectively analyzed for 30-day mortality and composite endpoints risk of complications and need for intervention using areas under the receiver-operating characteristics curve (AUROC). Subgroup analysis was conducted for patients with UGIB on admission and in-hospital UGIB.

**Results:**

A total of 252 patients were identified (67.5% men, mean age 63.8 ± 14.9 years). In-hospital UGIB occurred in 49.6%. AUROCs for 30-day mortality, risk of complications, and need for intervention (not applicable to RS) were 0.684 (95% confidence interval [CI]: 0.606–0.763), 0.665 (95% CI: 0.594–0.735), and 0.694 (95% CI: 0.612–0.775) for C-WATCH score, 0.724 (95% CI: 0.653–0.796) and 0.751 (95% CI: 0.687–0.815) for RS, 0.652 (95% CI: 0.57–0.735), 0.653 (95% CI: 0.579–0.727), and 0.673 (95% CI: 0.602–0.745) for p-RS and 0.652 (95% CI: 0.572–0.732), 0.663 (95% CI: 0.592–0.734), and 0.752 (95% CI: 0.683–0.821) for GBS. RS outperformed pre-endoscopic scores in predicting risk of complications, while there were no significant differences between pre-endoscopic scores except GBS outperforming p-RS in predicting need for intervention. The subgroup analysis obtained similar results. Positive predictive values for patients with estimated low risk for all three endpoints (C-WATCH score ≤1, RS ≤2, p-RS <1, and GBS ≤1) were 89%, 69%, 78%, and 92%.

**Conclusion:**

C-WATCH score performed similar to the established pre-endoscopic risk scores in patients with UGIB regarding relevant patient-related endpoints with no significant differences between both the subgroups.

## Introduction

Upper gastrointestinal bleeding (UGIB) is one of the leading indications for emergency endoscopy with an annual incidence of 36–172/100,000 [1, 2, 3]. Despite advances in medical and endoscopic treatment, it still is associated with a high mortality rate of up to 13% in patients presenting to the hospital with UGIB [4, 5, 6] and up to 26.5% in patients, who developed UGIB during their hospital stay [7, 8]. It can be subdivided into variceal and nonvariceal bleeding with peptic ulcers, esophagitis, and erosions being the most frequent causes of nonvariceal UGIB [9]. Early stratification of patients with UGIB according to their need for intervention, risk of complications, or death is reasonable to allocate patients to the appropriate medical and endoscopic treatment, e.g., urgent or elective endoscopy and in-hospital or out-of-hospital management, which subsequently may translate into saving costs and resources [10, 11]. The European Society of Gastrointestinal Endoscopy (ESGE) guideline on the diagnosis and management of nonvariceal upper gastrointestinal hemorrhage recommends the use of validated risk stratification tools to stratify patients into high- and low-risk groups [12]. Among these tools, the Glasgow-Blatchford score (GBS) which was developed to assess the need for treatment, and the Rockall score (RS) which was derived to assess mortality are the most widely evaluated and adopted [13, 14, 15]. In case of a low-risk situation, identified by a GBS of ≤1, ESGE and the corresponding guideline of the German Society of Gastroenterology, Digestive and Metabolic Diseases advise against early endoscopy or hospital admission with a very high sensitivity [12, 16]. The applicability of this cut-off value for the GBS was demonstrated by two multinational observational study, in which a GBS at a cut-off value of ≤1 had a specificity of 34.6% and 39.8%, respectively, and a positive predictive value (PPV) of 96.6% and 98.8%, respectively, for the identification of low-risk patients [17, 18]. In contrast to the GBS, the RS includes two endoscopic categories (diagnosis and major stigmata of recent hemorrhage) which are available after endoscopy and therefore limit its utility in the initial risk assessment [14]. Due to this limitation, a pre-endoscopic variant of the RS, also known as pre-endoscopic, admission, or initial RS, was also developed in the original study by Rockall et al. [14]. Based on a large nationwide observational study, Rockall et al. [19] suggested early endoscopy and discharge of patients with an RS ≤2. Despite their proven benefit in the setting of UGIB, risk assessment tools are used by only 51% of gastroenterologists according to a national survey, which was attributed to a lack of utility [20]. The new Cologne-WATCH (C-WATCH) score was designed as a pre-endoscopic score for acute variceal and nonvariceal UGIB and incorporates laboratory values only (c-reactive protein, white blood cell count, alanine-aminotransferase, thrombocytes, creatinine, and hemoglobin) with a minimum point value of 0 and a maximum point value of 8 [21]. Within the validation set, it predicted a composite endpoint consisting of recurrent bleeding, need for intervention (interventional radiology, surgery), or death within 30 days with an area under the receiver-operating characteristics curve (AUROC) of 0.704 [21]. About 38.7% of patients within the high-risk group, i.e., ≥2 points, reached the composite endpoint, whereas no patient classified as low risk (≤1 point) did [21]. Therefore, the aim of this study was to compare the C-WATCH score with the established risk scores of GBS, RS, and pre-endoscopic RS (p-RS) in patients with UGIB regarding 30-day mortality, risk of complications, and need for intervention.

## Methods

We retrospectively identified all patients who received an esophagogastroduodenoscopy (EGD) at the University Hospital Cologne, Department of Gastroenterology and Hepatology, between January 1 and December 31, 2017, from our endoscopy database. Inclusion criteria were age ≥18 years, presentation with signs and symptoms of acute UGIB (e.g., hematemesis, hematochezia, melena, or drop in hemoglobin concentration >2 g/dL in 24 h). Cases with incomplete data sets to compute GBS, RS, p-RS, and C-WATCH score or the composite endpoints were excluded (see online suppl. material; for all online suppl. material, see www.karger.com/doi/10.1159/000522121).

In accordance with the German law (paragraph 15, sentence 1, North Rhine Medical Association's professional code of conduct from November 14, 1998 as amended on November 19, 2011, and paragraph 6, sentence 1, Health Data Protection Act of North Rhine-Westphalia), approval by a local Ethics Committee, and written informed consent from the participants were not required because of the strictly retrospective design of our study.

The following data were retrieved from medical records: age, gender, arterial blood pressure, heart rate, signs, and symptoms of UGIB, comorbidities (see below), medication (antiplatelet drugs and anticoagulants), laboratory values (creatinine, blood urea, alanine-aminotransferase, c-reactive protein, white blood cell count, hemoglobin, thrombocytes), endoscopic findings, endoscopic, radiological, and surgical interventions for hemostasis, blood transfusion, rebleeding, and death within 30 days after index EGD. Vital status within 30 days after the index EGD was evaluated by contacting the appropriate civil registry offices.

Rebleeding was defined as endoscopic signs of UGIB within 30 days after initial hemostasis was obtained during index endoscopy. Liver diseases included but were not limited to acute and chronic viral hepatitis, alcoholic, and nonalcoholic fatty liver disease, autoimmune hepatitis, primary biliary cholangitis, primary sclerosing cholangitis, Wilson disease, and hemochromatosis. Major stigmata of recent hemorrhage were defined as blood in the upper gastrointestinal tract, an adherent clot, and a visible or spurting vessel [14]. Comorbidities included congestive heart failure, coronary artery disease, renal failure, liver disease, liver failure, disseminated malignancy, and any other major comorbidities.

C-WATCH score, RS, p-RS, and GBS were calculated as previously described [13, 14, 21], and compared regarding the following endpoints: (1) 30-day mortality, (2) composite endpoint “risk of complications” defined as rebleeding, need for interventional radiology or surgery to control bleeding and death within 30 days, derived from the original C-WATCH score study [21], and (3) composite endpoint “need for intervention” defined as use of blood transfusion or intervention (defined as endoscopic treatment, interventional radiology, or surgery), and rebleeding and death within 30 days in case of no intervention (not applicable to RS), derived from the original study by Blatchford et al. [13]. A subgroup analysis was performed for patients with UGIB on admission (subgroup A) and patients with in-hospital UGIB patients (subgroup B).

Low risk was defined as survival without reaching either composite endpoint during a 30-day follow-up. The following cut-off values were used to classify patients as low risk using according to previously published data [14, 18, 21]: C-WATCH score ≤1, RS ≤2, p-RS <1, and GBS ≤1. Furthermore, sensitivity, specificity, PPV, and negative predictive value (NPV) were calculated to assess risk scores performance in identifying patients at low risk.

Descriptive analysis was conducted using Statistical Package for the Social Sciences, version 27 (IBM, Armonk, NY, USA) and Medcalc (MedCalc Software, Ostend, Belgium). Categorical variables were presented as absolute and relative frequencies and analyzed by χ^2^ test. Continuous variables were expressed as means ± standard deviation, and compared using Student's *t* test. ROC curves were created and the AUROC with 95% confidence intervals was calculated. Delong test was used to compare AUROC for equality [22]. AUROCs of the same risk score were compared between both the subgroups based on the method by Hanley and McNeil [23]. Missing data were handled by using list-wise deletion. *p* values <0.05 were regarded as statistically significant, without any adjustment for multiple testing.

## Results

Among a total of 353 patients with UGIB, 101 (28.6%) cases were excluded because of incomplete data sets. There were no statistically significant differences between patients included in and excluded from the study regarding age, gender, intervention (endoscopic treatment, interventional radiology, surgery), rebleeding, and mortality, but excluded patients experienced in-hospital UGIB more frequently and had significantly less liver disease than patients included in our analysis (shown in online suppl. Table 1).

Among the remaining 252 patients, one-third were female (*n* = 82, 32.5%) and the mean age was 63.8 (±14.9) years. Melena (54%), hematemesis (40%), and hematochezia (18%) were the most frequent clinical signs of UGIB. The source of bleeding was classified as nonvariceal in 238 (94.4%) patients and variceal in only 14 (5.6%) patients. UGIB was the admission diagnosis in 127 (50.4%) cases (subgroup A), whereas UGIB occurred during the hospital stay in 125 (49.6%) cases (subgroup B). Further baseline characteristics are displayed in Table 1.

Results for all three endpoints are shown in Table 2. A total of 180 (71.4%) patients of the total cohort needed intervention and 77 (30.6%) patients experienced complications. The 30-day mortality amounted to 54 (21.4%). The proportion of patients with need for intervention and occurrence of complications were significantly higher in patients with in-hospital UGIB (78% vs. 65%, *p* = 0.031, and 40% vs. 21%, *p* = 0.001). Furthermore, more patients in this subgroup died within 30 days compared to patients with UGIB on admission (28% vs. 15%, *p* = 0.01).

**Fig. 1:**
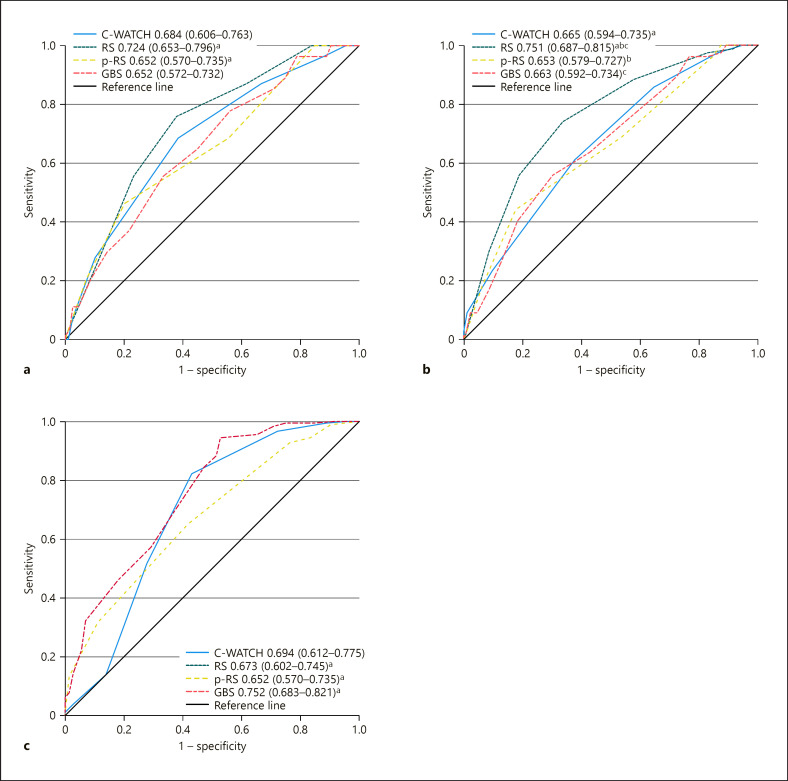
AUROCs of risk scores for the prediction of 30-day mortality (**a**), risk of complications (**b**), and need for intervention (**c**), not applicable to RS. Superscripted matching letters indicate statistical significances (*p* < 0.05) between marked risk scores. GBS, Glasgow-Blatchford score; p-RS, pre-endoscopic RS; RS, Rockall score.

The C-WATCH score, RS, p-RS, and GBS predicted 30-day mortality as well as both composite endpoints in the total cohort and in both subgroups with only moderate accuracy. In the total cohort, RS was significantly superior to p-RS regarding 30-day mortality and significantly superior to the C-WATCH score, p-RS, and GBS regarding the risk of complications. GBS outperformed p-RS regarding the need for intervention (shown in Table 3, Fig. 1). When comparing the risk scores within each subgroup, RS outperformed other scores in subgroup A and p-RS in subgroup B in predicting the risk of complications (shown in online suppl. Tables 2, 3). There were no differences in performances of each risk score in both subgroups except that RS predicted 30-day mortality more accurately in subgroup A (shown in Table 4).

During a 30-day follow-up, after the index EGD, 72 (28.6%) patients of the total cohort (44 [34.6%] and 28 [22.4%] patients of subgroups A and B) survived and did not meet either composite endpoint. Using the predefined cut-off values, 9 (3.6%), 16 (6.3%), 9 (3.6%), and 12 (4.8%) patients in the total cohort, comprising 6 (4.7%), 13 (10.2%), 8 (6.3%), and 8 (6.3%) patients in subgroup A and 3 (2.4%), 3 (2.4%), 1 (0.8%), and 4 (3.2%) patients in subgroup B were classified as low risk according to the C-WATCH score, RS, p-RS, and GBS. Of these, the C-WATCH score correctly identified 8 (88.9%), 5 (83.3%), and 3 (100%) low-risk patients in the total cohort, subgroup A and subgroup B, respectively. The corresponding numbers were 11 (68.8%), 10 (76.9%), and 1 (33.3%) patient for RS, 7 (77.8%), 6 (75%), and 1 (100%) patient for p-RS and were 11 (91.7%), 7 (87.5%), and 4 (100%) patients for GBS. Derived sensitivities, specificities, PPV, and NPV are displayed in Table 5.

## Discussion

In this monocentric retrospective study of patients with UGIB, the C-WATCH score performed equally to the established pre-endoscopic risk scores GBS and p-RS in predicting relevant patient-related outcomes, i.e., 30-day mortality, risk of complications, and need for intervention. Furthermore, it predicted 30-day mortality and need for intervention similar to RS. Similar results were observed in subgroup analysis of patients with UGIB on admission and UGIB during hospital stay. RS was most accurate in predicting the risk of complications in the total cohort and in patients with UGIB on admission. However, high sensitivities for the identification of patients at higher risk of an unfavorable outcome were accompanied by low specificities for all risk assessment tools investigated. In comparison to the established scores GBS, RS, and p-RS, the C-WATCH score may offer the advantage of simple calculation and its operator-independency as it relies on laboratory values only.

In the setting of UGIB, ESGE strongly recommends the use of a validated tool to stratify patients into high- and low-risk groups, since early risk stratification can assist clinical decision-making regarding the timing of endoscopy and hospital discharge [12]. A single risk stratification tool fails to cover all relevant aspects of risk assessment in this setting, limiting its applicability. This may be ascribed to the fact that these tools were originally derived to assess specific outcomes [12]. GBS was developed and validated to assess the need for treatment which was defined as the use of blood transfusion, any operative or endoscopic intervention to control the hemorrhage and death, rebleeding, or substantial fall in hemoglobin concentration after admission in case of no intervention [13]; RS was developed and validated to assess mortality [14]; the C-WATCH score was developed and validated to identify high-risk patients requiring urgent clinical management defined as recurrent bleeding, need for intervention, i.e., interventional radiology and surgery, or death within 30 days [21]. A systematic review including 16 studies reported on a substantial heterogeneity in outcomes and results between different risk scores [15]. We, therefore, used three endpoints to compare the C-WATCH score with RS, p-RS, and GBS of which the first composite endpoint corresponds to the endpoint of the C-WATCH score-trial and the second composite endpoint resembles the endpoint of the original study by Blatchford et al. [13, 21].

The evaluation of risk assessment tools in patients with UGIB has already been subject to numerous investigations [15, 18, 24, 25, 26]. Of these, the prospective international multicenter study conducted by Stanley et al. [18] represents one of the most recent and largest studies on this topic which included 3,012 consecutive patients with UGIB. In this study, GBS showed the highest AUROC (0.86) for intervention or death with a consistent performance in all participating countries. Furthermore, GBS at a cutoff ≤1 had a PPV of 96.6% to identify patients at a very low risk for intervention and death with a specificity of 34.6% [18]. Laursen et al. [17] investigated different cut-off values for GBS to identify low-risk patients. Cut-off values of 0, ≤1, and ≤2 had PPVs of 99.0%, 98.8%, and 96.9%, respectively, and specificities of 22.2%, 39.8%, and 48.9%. In our study, sensitivities and PPVs of all four risk scores investigated were of comparable magnitude. However, specificities were very low, ranging between 10% and 15% in the total cohort, and were even lower in the setting of in-hospital UGIB. Consequently, the majority of patients with low risk are not identified adequately, which reduces the efficacy of risk scoring during early risk stratification.

In our study, almost half of all cases with UGIB occurred in patients who were already hospitalized for another indication than UGIB. Previous studies from Germany and the USA found similar rates of in-hospital UGIB with 55% and 41% [7, 8]. In accordance with these studies, we also observed a significantly higher 30-day mortality in these patients. Although not statistically different, AUROCs of pre-endoscopic risk scores for 30-day mortality were numerically higher in patients with UGIB on admission compared to patients with in-hospital UGIB. Only the AUROC of RS was significantly higher in the former subgroup. This may be best explained by the hypothesis that mortality is determined mainly by other factors than UGIB in the setting of already hospitalized patients. In this regard, Haddad et al. [8] demonstrated that causes of mortality differed between both subgroups with a significantly higher mortality because of cardiovascular diseases, multiorgan failure, sepsis, and other reasons whereas mortality due to gastrointestinal bleeding was similar. In contrast to the study by Haddad et al. [8] who found no significant difference in GBS (12 vs. 13, *p* = 0.22) and RS (5 vs. 5, *p* = 0.45) between both the subgroups, more patients with in-hospital UGIB belonged to the high-risk RS category, i.e., RS ≥5, in the study by Klebl et al. [7]. We also observed higher median risk scores in patients with in-hospital UGIB compared to patients with UGIB on admission. Owing to low AUROCs of risk scores in the prediction of 30-day mortality and considerations about determinants of mortality in already hospitalized patients, we would argue against the regular use of risk scores to predict 30-day mortality in this scenario. In this regard, however, it needs to be accentuated that GBS and RS were derived from patients who were admitted because of UGIB and are therefore not validated for this scenario [13, 14]. A recently published randomized controlled trial challenged the use of GBS for early risk stratification and management allocation. In this study, urgent endoscopy, i.e., within 6 h, did not lead to a reduction in 30-day mortality compared to early elective endoscopy, i.e., between 6 and 24 h, in patients with GBS ≥12 [27]. Unfortunately, the impact of timing of endoscopy could not be considered in our study due to incomplete data.

Major limitations of our study derive from its retrospective and monocentric design. Although we did not observe significant differences between patients included in and excluded from analysis regarding age, gender, intervention, and mortality, one-quarter of initially eligible patients were excluded because of incomplete data sets, and hence selection bias cannot be ruled out, although it seems unlikely to substantially influence our results. In addition, we were not able to track courses of patients who did not receive EGD despite UGIB. We hypothesize that this would affect mainly low-risk patients and assume that this would most likely lead to an underestimation of NPV of risk assessment tools because of lower numbers of correctly classified low-risk patients, and it may explain the relatively high mortality rate observed in our study. Furthermore, patients with acute variceal bleeding accounted only for a minority of cases with UGIB which may limit the generalizability of our results in these patients. Other validated risk scores like AIMS65 (albumin level <30 g/dL, international normalized ratio >1.5, altered mental status, systolic blood pressure ≤90 mm Hg, and age >65 years) and Progetto nazionale emorragia digestive as well as the newly introduced ABC score were not considered for analysis mainly because of missing data sets to compute these risk scores and because of a lesser widespread use than GBS and RS [15, 28]. Lastly, we did not describe details on pharmacological intervention before endoscopy. The use of proton pump inhibitors (PPI) before endoscopy for suspected UGIB and its continuation in case of confirmed UGIB or findings during upper endoscopy susceptible for PPI pertains to the standard of care in these patients. Thus, it may be assumed that the vast majority of patients would have received PPI treatment.

## Conclusion

The simple-to-calculate and operator-independent C-WATCH score performed similar to the established risk scores RS, p-RS, and GBS in patients with acute UGIB in predicting the relevant patient-related endpoints 30-day mortality and need for intervention. Additionally, the C-WATCH score performed equally to the pre-endoscopic risk scores GBS and p-RS regarding the risk of complications. The efficacy of risk scores in terms of early risk stratification in patients with low risk may be limited due to their low specificity, albeit they exhibit good PPVs.

## Statement of Ethics

In accordance with the German law (paragraph 15, sentence 1, North Rhine Medical Association's professional code of conduct from November 14, 1998 as amended on November 19, 2011, and paragraph 6, sentence 1, Health Data Protection Act of North Rhine-Westphalia), approval by a local Ethics Committee and written informed consent from the participants were not required because of the strictly retrospective design of our study.

## Conflict of Interest Statement

Martin Bürger obtained consulting fees from Janssen and travel support from Pfizer. All other authors have no conflicts of interest to declare.

## Funding Sources

No funding has been received to conduct this study.

## Author Contributions

G.A., C.S., P.K., J.G., and M.B. conceived of the presented idea and designed the study. G.A., C.S., and J.G. retrieved data. G.A., J.F., and C.S. performed the analytic calculations. V.M., D.N., H.-M.S., and T.G. helped supervise the project. J.F., D.N., V.M., M.B., T.G., and H.-M.S. contributed to the interpretation of the results. G.A., C.S., and H.-M.S.: took the lead in writing the manuscript. All authors provided critical feedback and helped shape the research, analysis, and manuscript.

## Data Availability Statement

The data sets used and/or analyzed during the current study are available from the corresponding author on reasonable request.

## Supplementary Material

Supplementary data

Supplementary data

Supplementary data

## Figures and Tables

**Table 1:** Baseline characteristics of patients in the total cohort as well as patients with UGIB on admission (subgroup A) or during hospital stay UGIB (subgroup B)

	Total cohort	Subgroup A	Subgroup B	*p* value*
*n* (%)	252 (100)	127 (50.4)	125 (49.6)	
Men, *n* (%)	170 (67.5)	76 (59.8)	94 (75.2)	<0.01
Age, mean (±SD), years	63.8 (±14.9)	64.2 (±16.1)	63.3 (±13.6)	0.63
Signs of bleeding, *n* (%)				
Hematemesis	100 (39.7)	50 (39.4)	50 (40.0)	0.92
Hematochezia	45 (17.9)	24 (18.9)	21 (16.8)	0.66
Melaena	136 (54.0)	71 (55.9)	65 (52.0)	0.53
Syncope	26 (10.3)	10 (7.9)	16 (12.8)	0.2
Tachycardia	76 (30.2)	30 (23.6)	46 (36.8)	0.02
Systolic blood pressure, *n* (%)				
109–100 mm Hg	21 (8.3)	13 (10.2)	8 (6.4)	0.271
99–90 mm Hg	15 (6.0)	7 (5.5)	8 (6.4)	0.766
<90 mm Hg	46 (18.3)	13 (10.2)	33 (26.4)	0.001
Comorbidities, *n* (%)				
Liver disease	78 (31.0)	37 (29.1)	41 (32.8)	0.53
Heart failure	46 (18.3)	24 (18.9)	22 (17.6)	0.79
Renal failure	74 (29.4)	32 (25.2)	42 (33.6)	0.14
Metastatic malignancy	11 (4.4)	8 (6.3)	3 (2.4)	0.13
Medication, *n* (%)				
Anti-platelet drugs	58 (23.0)	23 (18.1)	35 (28.0)	0.06
Anticoagulants	61 (24.2)	33 (26.0)	28 (22.4)	0.51
Laboratory parameters, median (IQR), *n* (%)				
Hemoglobin, g/dL	8 (7–9.4)	8.1 (7–10.1)	8 (7–9)	0.02
White blood cells, µL^−1^	9.2 (6–13.6)	8.4 (5.5–12.7)	10.7 (7.2–14.4)	0.02
Thrombocytes, µL^−1^	197 (118–287)	192 (130–256)	199 (108–325)	0.86
Creatinine, mg/dL	1.3 (0.8–2.1)	1.1 (0.8–2)	1.6 (0.9–2.4)	0.25
Blood urea, mg/dL	68 (41–129)	63 (35–115)	74 (52–139)	0.15
ALT, U/L	22 (14–44.5)	19 (14–38)	24 (14–53)	0.08
CRP, mg/dL	22.1 (6.5–69.2)	9.2 (3.7–31.3)	49.7 (18.2–84.5)	<0.001
Endoscopic findings, *n* (%)				
Stigmata of recent hemorrhage	100 (39.7)	35 (27.6)	65 (52.0)	<0.001
Gastric ulcer	35 (13.9)	19 (15.0)	16 (12.8)	0.62
Duodenal ulcer	39 (15.5)	13 (10.2)	26 (20.8)	0.02
Erosive disease	37 (14.7)	17 (13.4)	20 (16.0)	0.56
Esophagitis	32 (12.7)	14 (11.0)	18 (14.4)	0.42
Malignancy	17 (6.7)	6 (4.7)	11 (8.8)	0.2
Variceal bleeding	14 (5.6)	11 (8.7)	3 (2.4)	0.03
Risk scores, mean (±SD)				
C-WATCH	4.1 (±1.4)	4.6 (±1.4)	3.7 (±1.3)	<0.001
f-RS	5.6 (±1.9)	6.2 (±1.8)	5.1 (±1.9)	<0.001
p-RS	3.8 (±1.5)	4.1 (±1.3)	3.5 (±1.6)	<0.001
GBS	10.9 (±4.3)	12.0 (±3.8)	9.8 (±4.4)	<0.001

ALT, alanine-aminotransferase; bpm, beats per minute; CRP, c-reactive protein; GBS, Glasgow-Blatchford score; IQR, interquartile range; p-RS, pre-endoscopic RS; RS, Rockall score; SD, standard deviation; SRH, stigmata of recent hemorrhage; UGIB, upper gastrointestinal bleeding; WBC, white blood cell count.

* Comparison between subgroups.

**Table 2:** Outcomes in patients of the total cohort as well as with UGIB on admission (subgroup A) and with in-hospital UGIB (subgroup B)

Outcome	Total cohort	Subgroup A	Subgroup B	*p* value*
30-day mortality	54 (21.4)	19 (15.0)	35 (28.0)	0.01
Risk of complications	77 (30.6)	27 (21.3)	50 (40.0)	0.001
Need for intervention	180 (71.4)	83 (65.4)	97 (77.6)	0.031
Rebleeding	38 (15.1)	12 (9.4)	26 (20.8)	0.01
Radiological intervention	6 (2.4)	0 (0)	6 (4.8)	0.01
Surgical intervention	5 (2.0)	1 (0.8)	4 (3.2)	0.17
Endoscopic intervention	59 (23.4)	26 (20.5)	33 (26.4)	0.27
Need for transfusion	164 (65.1)	74 (58.3)	90 (72.0)	0.02

Values are presented as *n* (%). UGIB, upper gastrointestinal bleeding.

* Comparison between subgroups.

**Table 3:** AUROC discrimination and comparison between scores regarding 30-day mortality, risk of complications, as well as need for intervention (not applicable to RS) in the total cohort

	AUROC and 95% CI 30-day mortality	C-WATCH*	RS*	p-RS*	GBS*
C-WATCH	0.684 (0.606–0.763)		0.44	0.58	0.44
RS	0.724 (0.653–0.796)	0.44		0.034	0.11
p-RS	0.652 (0.570–0.735)	0.58	0.034		0.99
GBS	0.652 (0.572–0.732)	0.44	0.11	0.99	

	**Risk of complications**	**C-WATCH** *	**RS** *	**p-RS** *	**GBS** *

C-WATCH	0.665 (0.594–0.735)		0.049	0.81	0.96
RS	0.751 (0.687–0.815)	0.049		<0.001	0.017
p-RS	0.653 (0.579–0.727)	0.81	<0.001		0.79
GBS	0.663 (0.592–0.734)	0.96	0.017	0.79	

	**Need for intervention**	**C-WATCH** *	**p-RS** *	**GBS** *	

C-WATCH	0.694 (0.612–0.775)		0.65	0.09	
p-RS	0.673 (0.602–0.745)	0.65		0.042	
GBS	0.752 (0.683–0.821)	0.09	0.042		

95% confidence interval in brackets. AUROC, area under the receiver-operating characteristic curve; GBS, Glasgow-Blatchford score; RS, Rockall score; p-RS, pre-endoscopic RS; n.a, not applicable.

* *p* values for comparison between scores.

**Table 4:** Area under the receiver-operating characteristic curve discrimination regarding 30-day mortality, risk of complications, and need for intervention (not applicable to RS) in patients with UGIB on admission (subgroup A) and with in-hospital UGIB (subgroup B)

	30-day mortality		*P* value*	Risk of complications		*p* value*	Need for intervention		*P* value*
	A	B		A	B		A	B	
C-WATCH	0.661 (0.531–0.792)	0.657 (0.553–0.761)	0.96	0.642 (0.532–0.753)	0.633 (0.534–0.732)	0.9	0.737 (0.638–0.836)	0.603 (0.467–0.74)	0.12
RS	0.815 (0.714–0.915)	0.63 (0.526–0.734)	0.013	0.783 (0.688–0.877)	0.701 (0.608–0.794)	0.23	n.a	n.a	
p-RS	0.727 (0.601–0.854)	0.584 (0.472–0.696)	0.1	0.679 (0.57–0.788)	0.618 (0.515–0.721)	0.43	0.709 (0.617–0.802)	0.608 (0.493–0.722)	0.17
GBS	0.713 (0.592–0.835)	0.575 (0.462–0.688)	0.1	0.671 (0.562–0.78)	0.619 (0.518–0.719)	0.49	0.768 (0.678–0.597)	0.711 (0.599–0.823)	0.43

GBS, Glasgow-Blatchford score; n.a. = not applicable, p-RS, pre-endoscopic RS; RS, Rockall score; UGIB, upper gastrointestinal bleeding.

* Comparison between subgroups; 95% confidence interval in brackets.

**Table 5:** Sensitivity, specificity, PPV, and NPV of risk scores using the combination of 30-day mortality, need for intervention, and risk of complications in the total cohort as well as patients with UGIB on admission (subgroup A) and with in-hospital UGIB (subgroup B)

	Cut-off	Sensitivity			Specificity			PPV			NPV		
		Total	A	B	Total	A	B	Total	A	B	Total	A	B
GBS	≤1	99.4	98.8	100	15.3	15.9	14.3	91.7	87.5	100	74.6	68.9	80.2
RS	≤2	97.2	96.4	97.9	15.3	22.7	3.6	68.8	76.9	33.3	74.2	70.2	77.9
p-RS	<1	98.9	97.6	100	9.7	13.6	3.6	77.8	75.0	100	73.3	68.1	78.2
C-WATCH score	≤1	99.4	98.8	100	11.1	11.4	10.7	88.9	83.3	100	73.7	67.8	79.5

A, subgroup A; B, subgroup B; GBS, Glasgow-Blatchford score; p-RS, pre-endoscopic Rockall score; RS, Rockall score; UGIB, upper gastrointestinal bleeding.
