# Supplementary material for: Comparison of Pre-Endoscopic C-WATCH Score with Established Risk Assessment Tools in Patients with Upper Gastrointestinal Bleeding
Source: Dig Dis. 2022 Jan 24;40(6):826–34. doi: 10.1159/000522121 (PMC9808639; doi:10.1159/000522121)
Supplement: Supplementary file 1 — Supplementary data [file ddi-0040-0826-s01.docx]

**Supplementary material**

**Table 1.** Comparison between patients included in and excluded from analysis.

|  | **included** | **excluded** | **p-value** |
| --- | --- | --- | --- |
| n (%) | 252 (71.4) | 101 (28.6) |  |
| Men, n (%) | 170 (67.5) | 62 (61.4) | 0.33 |
| Age [years], mean (±SD) | 63.8 (±14.9) | 66.2 (±17.1) | 0.21 |
| In-hospital UGIB, n (%) | 125 (49.6) | 75 (74.3) | <0.001 |
| Comorbidities, n (%) |  |  |  |
| Liver disease | 78 (31.0) | 18 (17.8) | 0.03 |
| Renal failure | 74 (29.4) | 26 (25.7) | 0.76 |
| Cardiac failure | 46 (18.3) | 15 (14.9) | 0.62 |
| Disseminated malignancy | 11 (4.4) | 7 (6.9) | 0.25 |
| Endoscopic findings, n (%) |  |  |  |
| Signs of hemorrhage | 100 (39.7) | 33 (32.7) | 0.22 |
| Gastric ulcer | 35 (13.9) | 13 (12.9) | 0.8 |
| Duodenal ulcer | 39 (15.5) | 10 (9.9) | 0.17 |
| Malignancy | 17 (6.7) | 2 (2.0) | 0.07 |
| Variceal bleeding | 14 (5.6) | 3 (3.0) | 0.31 |
| Laboratory parameters (±SD) |  |  |  |
| Hemoglobin [g/dL] | 8.6 (±2.3) | 8.7 (±2.5) | 0.55 |
| White blood cells [µL^-1^] | 11.1 (±8.6) | 10.6 (±8.2) | 0.65 |
| Thrombocytes [µL^-1^] | 223.1 (±162.8) | 252.1 (±152.7) | 0.14 |
| Creatinine [mg/dL] | 1.9 (±1.7) | 1.5 (±1.2) | 0.01 |
| Blood Urea [mg/dL] | 89.6 (±67.9) | 86.6 (±69.1) | 0.75 |
| ALT [U/l] | 44.9 (±127.1) | 44.8 (±65.1) | 0.99 |
| CRP [mg/dL] | 45.8 (±57.6) | 61.3 (73.5) | 0.08 |
| Signs of bleeding, n (%) |  |  |  |
| Hematemesis | 100 (39.7) | 32 (32.7) | 0.41 |
| Hematochezia | 45 (17.9) | 12 (11.9) | 0.29 |
| Melaena | 136 (54.0) | 45 (44.6) | 0.41 |
| Syncope | 26 (10.3) | 4 (4.0) | 0.07 |
| Tachycardia | 76 (30.2) | 13 (12.9) | 0.055 |
| Systolic blood pressure |  |  |  |
| <90 mmHg | 46 (18.3) | 8 (7.9) | 0.13 |
| Outcomes, n (%) |  |  |  |
| 30-day mortality | 54 (21.4) | 19 (18.8) | 0.78 |
| Rebleeding | 38 (15.1) | 12 (11.8) | 0.51 |
| Any endoscopic, surgical or radiological intervention | 63 (25.0) | 18 (17.8) | 0.26 |
| Need for Transfusion | 164 (65.1) | 53 (54.5) | 0.14 |
|  |  |  |  |

Intervention defined as endoscopic treatment, interventional radiology and surgery.

Abbreviatons: ALT=alanine aminotransferase; CRP=C-reactive protein; SD=standard deviation.
