# Supplementary material for: Comparison of Pre-Endoscopic C-WATCH Score with Established Risk Assessment Tools in Patients with Upper Gastrointestinal Bleeding
Source: Dig Dis. 2022 Jan 24;40(6):826–34. doi: 10.1159/000522121 (PMC9808639; doi:10.1159/000522121)
Supplement: Supplementary file 2 — Supplementary data [file ddi-0040-0826-s02.docx]

**Supplementary material**

**Table 2.** AUROC discrimination and comparison between scores regarding 30 day-mortality, risk of complications as well as need for intervention (not applicable to RS) in patients with UGIB on admission (subgroup A).

|  | **AUROCs and 95% CI** |  |  |  | |  | |
| --- | --- | --- | --- | --- | --- | --- | --- |
|  | **30-day mortality** | **C-WATCH score*** | **RS*** | **p-RS*** | | **GBS*** | |
| **C-WATCH score** | 0.661 (0.531 – 0.792) |  | 0.07 | 0.47 | | 0.49 | |
| **RS** | 0.815 (0.714 – 0.915) | 0.07 |  | 0.14 | | 0.09 | |
| **p-RS** | 0.727 (0.601 – 0.854) | 0.47 | 0.14 |  | | 0.84 | |
| **GBS** | 0.713 (0.592 – 0.835) | 0.49 | 0.09 | 0.84 | |  | |
|  | **Risk of complications** | **C-WATCH score*** | **RS*** | **p-RS*** | | **GBS*** | |
| **C-WATCH score** | 0.642 (0.532 – 0.753) |  | 0.04 | 0.6 | | 0.61 | |
| **RS** | 0.783 (0.688 – 0.877) | 0.04 |  | 0.038 | | 0.039 | |
| **p-RS** | 0.679 (0.57 – 0.788) | 0.6 | 0.038 |  | | 0.89 | |
| **GBS** | 0.671 (0.562 – 0.78) | 0.61 | 0.039 | 0.89 | |  | |
|  | **Need for Intervention** | **CWATCH score*** | **p-RS*** | | **GBS*** | |  |
| **C-WATCH score** | 0.737 (0.638 – 0.836) |  | 0.6 | | 0.47 | |  |
| **p-RS** | 0.709 (0.617 – 0.802) | 0.6 |  | | 0.24 | |  |
| **GBS** | 0.768 (0.678 – 0.597) | 0.47 | 0.24 | |  | |  |

*Comparison between scores; 95%-confidence interval in brackets.

Abbreviations: AUROC= Area under the receiver-operating characteristic curve; GBS= Glasgow Blatchford Score; n.a.=not applicable; p-RS= pre-endoscopic RS; RS= Rockall score, UGIB= upper gastrointestinal bleeding.
