# Supplementary material for: Comparison of Pre-Endoscopic C-WATCH Score with Established Risk Assessment Tools in Patients with Upper Gastrointestinal Bleeding
Source: Dig Dis. 2022 Jan 24;40(6):826–34. doi: 10.1159/000522121 (PMC9808639; doi:10.1159/000522121)
Supplement: Supplementary file 3 — Supplementary data [file ddi-0040-0826-s03.docx]

**Supplementary material**

**Table 3.** AUROC discrimination and comparison between scores regarding 30 day-mortality, risk of complications as well as need for intervention (not applicable to RS) in patients with in-hospital UGIB

(Subgroup B).

|  | **AUROCs and 95% CI** |  |  |  | |  | |
| --- | --- | --- | --- | --- | --- | --- | --- |
|  | **30-day mortality** | **C-WATCH score*** | **RS*** | **p-RS*** | | **GBS*** | |
| **C-WATCH score** | 0.657 (0.553 – 0.761) |  | 0.71 | 0.34 | | 0.14 | |
| **RS** | 0.63 (0.526 – 0.734) | 0.71 |  | 0.27 | | 0.43 | |
| **p-RS** | 0.584 (0.472 – 0.696) | 0.34 | 0.27 |  | | 0.89 | |
| **GBS** | 0.575 (0.462 – 0.688) | 0.14 | 0.43 | 0.89 | |  | |
|  | **Risk of complications** | **C-WATCH score*** | **RS*** | **p-RS*** | | **GBS*** | |
| **C-WATCH score** | 0.633 (0.534 – 0.732) |  | 0.28 | 0.83 | | 0.8 | |
| **RS** | 0.701 (0.608 – 0.794) | 0.28 |  | 0.02 | | 0.13 | |
| **p-RS** | 0.618 (0.515 – 0.721) | 0.83 | 0.02 |  | | 0.99 | |
| **GBS** | 0.619 (0.518 – 0.719) | 0.8 | 0.13 | 0.99 | |  | |
|  | **Need for Intervention** | **CWATCH score*** | **p-RS*** | | **GBS*** | |  |
| **C-WATCH score** | 0.603 (0.467 – 0.74) |  | 0.96 | | 0.09 | |  |
| **p-RS** | 0.608 (0.493 – 0.722) | 0.96 |  | | 0.13 | |  |
| **GBS** | 0.711 (0.599 – 0.823) | 0.09 | 0.13 | |  | |  |

*Comparison between scores; 95%-confidence interval in brackets.

Abbreviations: AUROC= Area under the receiver-operating characteristic curve; GBS= Glasgow Blatchford Score; n.a.=not applicable; p-RS= pre-endoscopic RS; RS= Rockall score.
